# Differential expression profile of circular RNAs in mouse peritoneum with peritoneal fibrosis and the potential regulatory role of novel_circ_0007527

**DOI:** 10.1016/j.gendis.2023.04.025

**Published:** 2023-06-23

**Authors:** Yingfeng Shi, Yan Hu, Hui Chen, Jinqing Li, Min Tao, Xun Zhou, Qin Zhong, Andong Qiu, Shougang Zhuang, Na Liu

**Affiliations:** a Department of Nephrology, Shanghai East Hospital, Tongji University School of Medicine, Shanghai 200120, China; b School of Life Science and Technology, Advanced Institute of Translational Medicine, Tongji University, Shanghai 200092, China; c Department of Medicine, Rhode Island Hospital and Alpert Medical School, Brown University, Providence, RI 02912, USA

CircRNAs, a novel type of endogenous non-coding RNAs (ncRNAs), are typically produced by back-splicing from exons of protein-coding genes, which are characterized by a covalently closed structure with neither 5′ to 3′ polarity nor a poly(A) tail.^1^ On the one hand, circRNAs are involved in the regulation of a variety of important biological processes, such as apoptosis, proliferation, migration, and inflammatory responses.^1^ On the other hand, the relationship between circRNAs and several fibrotic diseases has been reported.^2^ However, the functions of circRNAs in peritoneal fibrosis (PF) are still unknown. Herein, we first reported the circRNA expression profile in the high glucose peritoneal dialysis fluid (HG-PDF)-induced PF mouse model, and the differentially expressed circRNAs between the PDF group and Sham group. The Gene ontology (GO) enrichment and Kyoto Encyclopedia of Genes and Genomes (KEGG) analysis and the construction of circRNA–miRNA–mRNA network provided an overview of the role of circRNAs in the development of PF. In addition, novel_circ_0007527 was up-regulated in the *in vitro* model of injured peritoneal mesothelial cells. Knockdown of novel_circ_0007527 inhibited peritoneal mesothelial epithelial–mesenchymal transition (EMT), proliferation, migration, and apoptosis. Therefore, our study provides novel avenues for PF research from the view of circRNAs.

We established a PF mouse model by intraperitoneal injection with 4.25% HG-PDF for 28 days and then collected parietal peritoneum tissues from each mouse in the Sham group and PDF group. The histopathological examination in Masson's trichrome staining and Sirius red staining indicated obvious thickening fibrotic peritoneum, which suggested that the PF mouse model was successfully constructed (Fig. S1). To detect the expression profile of circRNAs in PF, high-throughput whole transcriptome sequencing analyses were conducted in peritoneal tissues from both the Sham group and the PDF group. A total of 10,356 circRNAs were detected from two groups. Of them, 432 circRNAs were aberrantly expressed in the fibrotic peritoneum (*P* < 0.05, |log_2_fold change| > 1), with 229 up-regulated circRNAs and 203 down-regulated circRNAs (Fig. 1A). The top 8 up-regulated and down-regulated circRNAs were demonstrated in the heat map in Figure 1A. To better understand the features of differentially expressed circRNAs in the fibrotic peritoneum, detailed information on the top 10 up-regulated circRNAs and top 10 down-regulated circRNAs are listed in Tables S1 and 2. The GO enrichment and KEGG pathway enrichment are provided in Figure S3 and 4.

**Figure 1:**
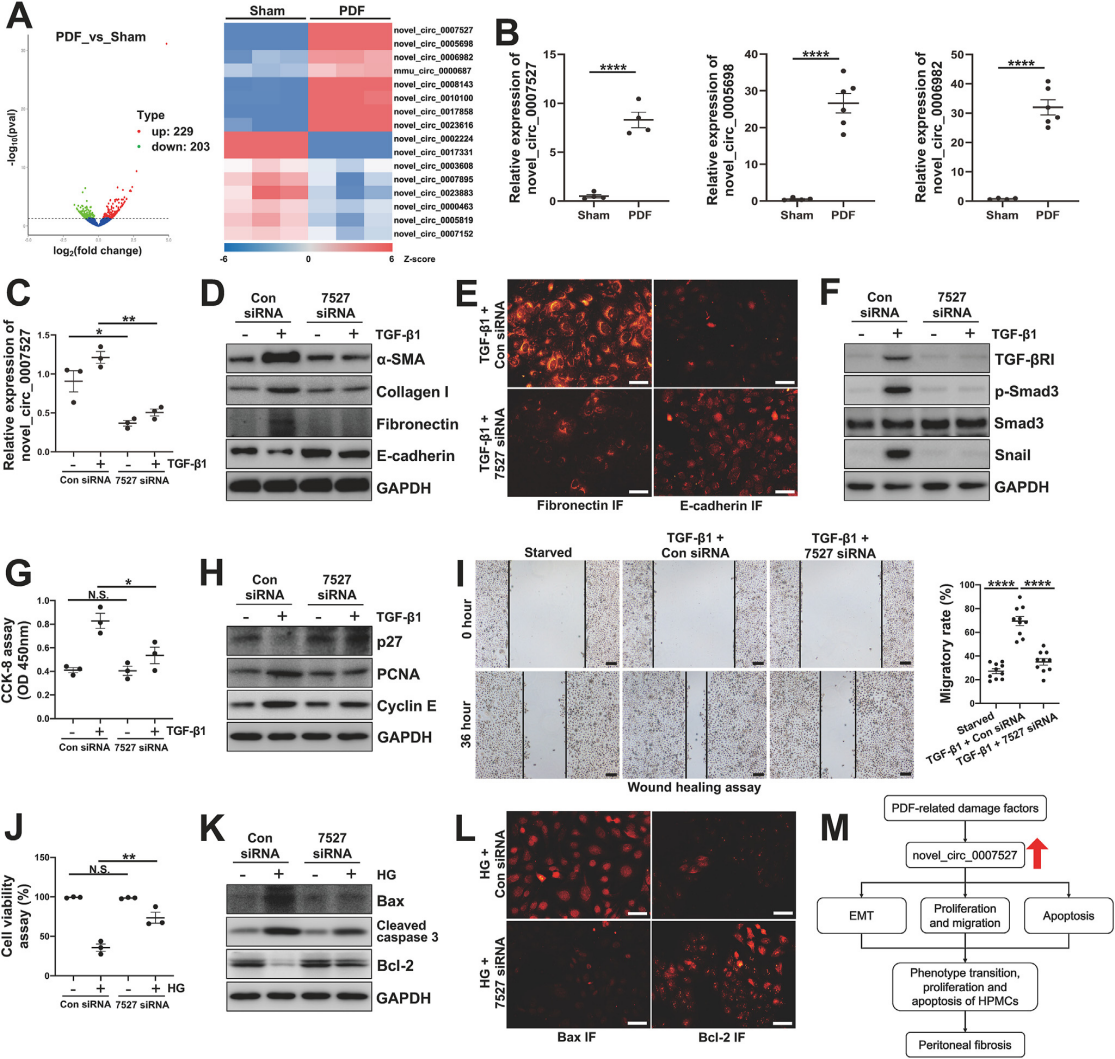
Expression profile of circRNAs in mouse peritoneum with peritoneal fibrosis and the potential role of novel_circ_0007527 in promoting peritoneal mesothelial epithelial–mesenchymal transition, proliferation, migration, and apoptosis. **(A)** High-throughput whole transcriptome sequencing and subsequent bioinformatics analysis were performed by Novogene Bioinformatics Technology in the Sham group and PDF group. Volcano plot of differentially expressed circRNAs in the two groups with the up-regulated circRNAs in red and down-regulated circRNAs in green (*P* < 0.05, |log_2_fold change| > 1). Hierarchical cluster analysis of top 8 up-regulated and down-regulated circRNAs in peritoneal tissues. **(B)** Three selected circRNAs were confirmed by qRT-PCR in peritoneum tissues from the Sham group and PDF group. **(C)** Serum-starved HPMCs were transfected with siRNA targeting novel_circ_0007527 or scrambled siRNA and then incubated with TGF-β1 (2 ng/mL) for an additional 36 h. The expression of novel_circ_0007527 was validated by qRT-PCR. **(D)** Western blot analysis was performed to evaluate the expression of α-SMA, collagen I, fibronectin, E-cadherin, and GAPDH in HPMCs cell lysates. **(E)** The photomicrographs illustrating the immunofluorescence of fibronectin and E-cadherin. **(F)** Western blot analysis was performed to evaluate the expression of TGF-βRI, p-Smad3, Smad3, Snail, and GAPDH in HPMCs cell lysates. **(G)** The results from the CCK-8 assay demonstrating the cell proliferation induced by TGF-β1 in HPMCs. **(H)** Western blot analysis was performed to evaluate the expression of p27, PCNA, cyclin E, and GAPDH in HPMCs cell lysates. **(I)** Wound healing assay and quantitative analysis were conducted to evaluate the migration ability of HPMCs. **(J)** Serum-starved HPMCs were transfected with siRNA targeting novel_circ_0007527 or scrambled siRNA and then incubated with 4.25% HG-PDF (1:1 mixture of MEM and HG-PDF) for an additional 36 h. Cell viability was evaluated by CCK-8 assay. **(K)** Western blot analysis was performed to evaluate the expression of Bax, cleaved caspase 3, Bcl-2, and GAPDH in HPMCs cell lysates. **(L)** The photomicrographs illustrating the immunofluorescence of Bax and Bcl-2. **(M)** Mechanism diagram of novel_circ_0007527 regulating peritoneal fibrosis. Data were represented as mean ± standard error of the mean. ^∗^*P* < 0.05, ^∗∗^*P* < 0.01, ^∗∗∗∗^*P* < 0.0001. N.S., not statistically significant. Scale bars in (E) and (L) were 50 μm, and scale bars in (I) were 500 μm.

Multiple studies have demonstrated that circRNAs regulate gene expression by acting as “miRNA sponges”. To better explore the potential function of the differentially expressed circRNAs in PD-related PF, we used the TargetScan database to predict the miRNA binding sites on the circRNA. The top 5 miRNAs for the most differential up-regulated circRNAs and the top 5 mRNAs for each miRNA were displayed as a circRNA–miRNA–mRNA network using Cytoscape software (version 3.8.2). As demonstrated in Figure S2, the different circRNA (red hexagon) might have a common binding site of miRNA (green triangles) and mRNA (purple oval). Detailed information on the circRNA-miRNA-mRNA network was listed in Tables S3 and 4. Many associated mRNAs played a crucial role in the development of fibrotic diseases, such as Egf, Ezh2, Wnt, Jun, and Twist.

In order to identify the results from high-throughput sequencing, we selected the three most up-regulated circRNAs for verification by qRT-PCR based on fold changes. The results indicated that the expression levels of novel_circ_0007527, novel_circ_0005698, and novel_circ_0006982 were significantly up-regulated in the PDF group compared with that in the Sham group (Fig. 1B). The expression trends of these three circRNAs validated by qRT-PCR were consistent with the high-throughput sequencing analysis. Among the three circRNAs, the *p*-value of novel_circ_0007527 was the most significant (Table S1). Therefore, we selected novel_circ_0007527 for further functional analysis in an *in vitro* model. As shown in Figure 1C, following stimulation with TGF-β1 at 2 ng/mL, novel_circ_0007527 expression was significantly increased, which was detected by qRT-PCR. This result indicated that novel_circ_0007527 might play a pivotal role in TGF-β1-injured peritoneal mesothelial cells. Immunoblotting and immunofluorescence results demonstrated that TGF-β1 induced EMT of human peritoneal mesothelial cells (HPMCs), while transfection with novel_circ_0007527 siRNA significantly decreased the expression of α-SMA, collagen I, and fibronectin, and increased the protein level of E-cadherin (Fig. 1D, E). Many profibrotic pathways, such as the TGF-β/Smad3 signaling pathway, are closely related to the process of EMT.^3^ We thus evaluated whether novel_circ_0007527 participated in the process of EMT via mediating the TGF-β/Smad3 signaling pathway. As shown in Figure 1F, the knockdown of novel_circ_0007527 significantly decreased the expression of TGF-β receptor I (TGF-βRI) and phosphorylation of Smad3 (p-Smad3) and reduced EMT-associated transcriptional factors, such as Snail. Taken together, these data suggested that novel_circ_0007527 might play a pivotal role in the development of PF, and the knockdown of novel_circ_0007527 inhibited TGF-β1-induced EMT by regulating the TGF-β/Smad3 signaling pathway.

It is well known that HPMCs undergoing EMT would lose tight junctions between adjacent cells and acquire the ability of cell proliferation and migration.^4^ To examine whether novel_circ_0007527 participated in the process of proliferation, we conducted a CCK-8 assay in HPMCs. The result indicated that TGF-β1 substantially stimulated cell proliferation, while novel_circ_0007527 knockdown blocked the proliferation of HPMCs (Fig. 1G). Immunoblotting and immunofluorescence analysis demonstrated that the knockdown of novel_circ_0007527 significantly increased the protein level of p27 and decreased levels of PCNA and cyclin E (Fig. 1H; Fig. S5), which were essential regulatory proteins related to cell proliferation. In addition, wound healing assay revealed that TGF-β1 stimulation increased the migratory rate of HPMCs, while knockdown of novel_circ_0007527 suppressed the migration ability (Fig. 1I). Collectively, novel_circ_0007527 knockdown inhibited TGF-β1-induced proliferation and migration of HPMCs.

To detect whether 4.25% HG-PDF affected the cell viability of HPMCs, CCK-8 assays were performed. As shown in Figure 1J and 4.25% HG-PDF significantly decreased the cell viability of the HPMCs, while novel_circ_0007527 knockdown reversed this phenomenon. Immunoblot analyses and immunofluorescence staining demonstrated that 4.25% HG-PDF largely increased the expression of Bax and cleaved caspase 3, and substantially decreased the apoptosis-suppressor protein Bcl-2. Knockdown of novel_circ_0007527 reduced the expression levels of Bax and cleaved caspase 3 and reversed the expression of Bcl-2 (Fig. 1K, L). Taken together, these data suggested that novel_circ_0007527 knockdown inhibited high glucose-induced human peritoneal mesothelial cell apoptosis.

To our knowledge, circRNAs are promising as treatment targets. A previous study indicated that circRNAs provide novel insight to design innovative therapeutic strategies for cancer treatment.^5^ For instance, CiRS-7/cdr1as is up-regulated and mediates oesophageal squamous cell carcinoma, gastric cancer, colorectal cancer, and hepatocellular carcinoma by different pathways, which suggests that the decreased expression of CiRS-7/Cdr1as might offer an effective treatment method. In addition, circRNAs are generally enriched and stable in the cytoplasm, indicating that they might be detected non-invasively in bodily fluids and more easily accepted by patients. Thus, circRNAs are potential biomarkers for many diseases. Therefore, in-depth studies on the relationship between circRNAs and the mechanism of PF will help discover promising biomarkers for disease diagnosis and treatment.

Collectively, we analyzed the difference of circRNAs in peritoneum tissue between the Sham group and PDF group and found a variety of circRNAs related to peritoneal dialysis-associated PF. Furthermore, we demonstrated that the knockdown of novel_circ_0007527 acted an important role in the development of PF. The anti-fibrotic role of novel_circ_0007527 knockdown may ascribe to the inhibition of peritoneal EMT, proliferation, migration, and apoptosis (Fig. 1M). The novel_circ_0007527 might contribute to the peritoneal EMT by regulating the TGF-β/Smad3 signaling pathway. Thus, our study provides novel avenues for PF research and understanding the potential relationship between circRNAs and PF.

## Author contributions

N.L. participated in the research design. Y.S., Y.H., H.C., J.L., M.T., X.Z., and Q.Z. conducted experiments. Y.S., Y.H., H.C., A.Q., S.Z., and N.L. contributed new reagents or analytic tools. Y.S., Y.H., H.C., and N.L. performed data analysis. Y.S., Y.H., H.C., N.L., S.Z., and A.Q. wrote or contributed to the writing of the manuscript. All authors approved the final version of the manuscript.

## Conflict of interests

No conflict of interests, financial or otherwise, is declared by the author(s).

## Funding

This study was supported by grants from the National Nature Science Foundation of China (No. 82070791, 81670690, 81470991, 81200492 to N.L.; No. 82070700, 81830021 to S.Z.), the Shanghai Scientific Committee of China (No. 20ZR1445800, 23ZR1452200, 13PJ1406900 to N.L.), the Outstanding Leaders Training Program of Pudong Health Bureau of Shanghai, China (No. PWR12021-02 to N.L.), the Shanghai Health Bureau and Shanghai Administration of Traditional Chinese Medicine of China (No. ZHYY-ZXYJHZX-202114 to N.L.), the Project of Pudong Health Bureau of Shanghai, China (No. PW2021D-04, PWZxk2022-05, PWZxk2017-05 to N.L.), the Clinical Investigation Grant of Shanghai East Hospital, Shanghai, China (No. DFLC2022016 to N.L.), the Shanghai Sailing Program (China) (No. 23YF1434700 to Y.S.), the China Postdoctoral Science Foundation (No. 2021M692436 to Y.S.), and the Branch Grant of National Key R&D Program of China (No. 2018YFA0108802 to S.Z.).
