# Supplementary material for: Differential expression profile of circular RNAs in mouse peritoneum with peritoneal fibrosis and the potential regulatory role of novel_circ_0007527
Source: Genes Dis. 2023 Jun 23;11(3):100991. doi: 10.1016/j.gendis.2023.04.025 (PMC10806300; doi:10.1016/j.gendis.2023.04.025)
Supplement: Multimedia component 1 [file mmc1.docx]

**Differential expression profile of circular RNAs in mouse peritoneum with peritoneal fibrosis and the potential regulatory role of novel_circ_0007527**

Yingfeng Shi^a,1^, Yan Hu^a,1^, Hui Chen^a,1^, Jinqing Li^a^, Min Tao^a^, Xun Zhou^a^, Qin Zhong^a^, Andong Qiu^b^, Shougang Zhuang^a,c^, Na Liu^a,^*

**Supplementary Materials**

This file includes:

1. Supplementary Materials and Methods

2. Supplementary Figures

3. Supplementary Tables

**1. Supplementary Materials and Methods**

**Antibodies and reagents**

TGF-β1 protein was purchased from R&D Systems (Minneapolis, MN, United States). Antibodies to Collagen I (sc-28654), TGF-βRI (sc-399), GAPDH (sc-32233), and PCNA (sc-71858) were purchased from Santa Cruz Biotechnology (San Diego, CA, United States). Antibody to Fibronectin (ab2413) was purchased from Abcam (Cambridge, MA, United States). Antibodies to E-cadherin (#14472), p-Smad3 (#9520), Smad3 (#9523), Snail (#3879), p27 (#2552), Cyclin E (#20808), and Cleaved caspase 3 (#9664) were purchased from Cell Signaling Technology (Danvers, MA, United States). Antibody to Bax (556467) was purchased from BD Bioscience (San Diego, CA). Antibody to Bcl-2 (BS1511) was purchased from Bioworld Technology (Nanjing, China). Small interfering (si)RNA-novel_circ_0007527 and siRNA-negative control were purchased from GenePharma (Shanghai, China). Lipofectamine 2000 was purchased from Invitrogen (Grand Island, NY, United States). The Cell Counting Kit-8 (CCK-8) proliferation assay kit was purchased from Beyotime Biotechnology (Shanghai, China). 4.25% glucose PDF was purchased from Baxter Healthcare (Guangzhou, China). Antibody to α-SMA (A2547) and all other chemicals were obtained from Sigma-Aldrich (St. Louis, MO, United States).

**Animal model and experimental design**

Male C57/black mice (Shanghai Super-B&K Laboratory Animal Corp. Ltd, Shanghai, China) that weighed 20-25g were housed under a 12h light-dark cycle with food and water supplied ad libitum. All animal work was performed in Tongji University school of medicine (Shanghai, China). The mouse model of peritoneal fibrosis was established by daily intraperitoneal injection of 100 ml/kg 4.25% high glucose peritoneal dialysis fluid (Baxter Healthcare, Guangzhou, China) for 28 days.^1,2^ Mice were randomly divided into two groups with 6 mice per group: (1) mice injected with an equivalent amount of saline intraperitoneally (Sham group); (2) mice injected with 100 ml/kg 4.25% high glucose peritoneal dialysis fluid intraperitoneally (PDF group). At the end of 28 days, all mice were killed by exsanguination under anesthesia with inhaled 5% isoflurane in room air and the parietal peritoneum was collected from each mouse for further histological examination and High‐throughput whole transcriptome analysis. Animal procedures were approved by the Institute’s Animal Ethics Committee of Tongji University (Shanghai, China) (TJBB00623108).

**Morphologic studies of peritoneum**

Formalin-fixed peritoneum was embedded in paraffin and cut into 3-μm-thick sections. To detect the peritoneal fibrosis, Masson’s trichrome staining and Sirius red staining were performed according to the protocol provided by the supplier (Sigma-Aldrich). The thickness and positive area of the submesothelial tissue were measured, and the average of ten independent measurements was calculated for each section (original magnification, ×200).

**High‐throughput sequencing and analysis of circRNAs**

High‐throughput whole transcriptome sequencing and subsequent bioinformatics analysis were performed by Novogene Bioinformatics Technology (Beijing, China) as previously reported.^3^

**Construction of circRNA-miRNA-mRNA interaction network**

We constructed the circRNA-miRNA-mRNA network by using Cytoscape software, which is an online bioinformatics resource platform for establishing biomolecular interaction networks. For each circRNA, five miRNAs most likely binding to the circRNA and top 5 target genes to every miRNA were predicted.

**Quantitative real-time polymerase chain reaction (qRT-PCR) validation**

qRT-PCR was used to validate the top 3 up-regulated circRNAs expression level. Total RNA was isolated from the peritoneum of PDF group and Sham group using TRIzol reagent (Invitrogen, CA, USA). A reverse transcriptase kit (abmgood, Shanghai, China) was used to synthesize cDNA, and qRT-PCR analysis was conducted in ABI 7500 real-time detection system by using Blas Taq (abmgood, Shanghai, China). The primer sequences were as follows. novel_circ_0007527, forward: 5'-GGCAGTGGCTCTCAAGTGGATTC-3', reverse: 5'-GACCAAAGGCTGCATTCACAAAGC-3', novel_circ_0005698, forward: 5'-GGACCAACTTCTCAGCCCAACAG-3', reverse: 5'-CTTGGCCTGTCTAGCAGCATCG-3', novel_circ_0006982, forward: 5'-CCAGCCAAGCACCAATACAGAACC-3', reverse: 5'-CCCCAGCAGAGAGCCACCAG-3', and the internal control GAPDH, forward: 5'-GGTTGTCTCCTGCGACTTCA-3', reverse: 5'-TGGTCCAGGGTTTCTTACTCC-3'. Each group were set up triplicated wells and three experimental repeats.

**Cell culture and siRNA transfection**

Human peritoneal mesothelial cells (American Type Culture Collection ATCC; Rockville, MD) were cultured in MEM medium containing 10% fetal bovine serum (FBS), 1% penicillin and streptomycin in an atmosphere of 5% CO_2_ and 95% air at 37°C. After passing the primary HPMCs for three generations, we obtained a stable phenotype to start the formal experiments. HPMCs were grown in 6-well plates until they reached 60-70% confluence before transfection. siRNA-novel_circ_0007527 and siRNA-negative control were transiently transfected into HPMCs with Lipofectamine 2000 as previously reported.^4^ The sequence of novel_circ_0007527 siRNA is 5'-GAACAAGGACCAUGGGUUUTT-3' (sense strand), 5'-AAACCCAUGGUCCUUGUUCTT-3' (antisense strand). After 24 hours transfection, the cells were incubated with/without TGF-β1 (2 ng/ml) or 4.25% HG-PDF (1:1 mixture of MEM and HG-PDF) for additional 36 hours. Finally, cell samples were harvested for further experiment. All of the in vitro experiments were repeated at least three times.

**Western blot**

Total protein was extracted from the HPMCs, the western blot analysis was performed as described previously.^5^ The gray values of immunoblot results were calculated by using Image J software (National Institutes of Health, Bethesda, MD, USA).

**Immunofluorescence staining**

The cells grown on chamber glides were subjected to 4% formaldehyde for 15 min to immobilization. Next, the HPMCs were permeabilized by adding 0.1% Triton X-100 for 15 min. The HPMCs were blocked with 10% goat serum for 30 min. Then, antibodies to Fibronectin, E-cadherin, PCNA, Bax, and Bcl-2 were added respectively and incubated overnight at 4℃, followed by Texas Red-labeled secondary antibody for 1 hour at room temperature in the dark. The nuclei were counterstaining with DAPI for 10 min. Finally, images were photographed with an Olympus fluorescence microscope.

**CCK-8 assay**

The CCK-8 assay was conducted to evaluate cell proliferation and cell viability according to the manufacture's protocols. Briefly, HPMCs were seed into a 96-well plate. After the process of siRNA transfection, the original culture medium was removed, and added 100μl fresh medium containing 10μl CCK-8 reagent to each well for an additional 4 hours, then measured the absorbance using a microplate reader at 450 nm.

**Wound healing assay**

Wound healing assay was conducted to evaluate cell migration. Briefly, HPMCs from different groups were seeded into a 6-well plate and incubated at 37℃ until 90% confluence. A micropipette tip was used to create a scratch wound, and the scratched-off HPMCs were cleared by PBS in three times. Next, the HPMCs were incubated in serum-free MEM with TGF-β1 (2 ng/ml) in the presence or absence of novel_circ_0007527 siRNA. The migrating cells were photographed at 0 and 36 hours under an inverted microscope. The width of the wound was analyzed using Image J software (National Institutes of Health, Bethesda, MD, USA). The migratory rate was calculated as (A−B)/A×100%, where A and B represent the width of the wound at 0 and 36 hours, respectively.

**Statistical analysis**

Data depicted in graphs represent the means ± SEM for each group. Intergroup comparison was made using one-way ANOVA, multiple means were compared using Tukey’s test, the differences between two groups were determined by Student’s t-test. Statistically significant differences between mean values were marked in each graph. *P*<0.05 was considered statistically significant. The statistical analyses were conducted by using IBM SPSS Statistics 20.0 (Beijing, China).

**Supplementary references**

1. Xu L, Liu N, Gu H, et al. Histone deacetylase 6 inhibition counteracts the epithelial-mesenchymal transition of peritoneal mesothelial cells and prevents peritoneal fibrosis. *Oncotarget.* 2017;8(51):88730-88750.

2. Shi Y, Tao M, Wang Y, et al. Genetic or pharmacologic blockade of enhancer of zeste homolog 2 inhibits the progression of peritoneal fibrosis. *J Pathol.* 2020;250(1):79-94.

3. Zhou CX, Ai K, Huang CQ, et al. miRNA and circRNA expression patterns in mouse brain during toxoplasmosis development. *BMC Genomics.* 2020;21(1):46.

4. Shi Y, Tao M, Ni J, et al. Requirement of Histone Deacetylase 6 for Interleukin-6 Induced Epithelial-Mesenchymal Transition, Proliferation, and Migration of Peritoneal Mesothelial Cells. *Front Pharmacol.* 2021;12:722638.

5. Hu Y, Shi Y, Chen H, et al. Blockade of Autophagy Prevents the Progression of Hyperuricemic Nephropathy Through Inhibiting NLRP3 Inflammasome-Mediated Pyroptosis. *Frontiers in Immunology.* 2022;13.

**2. Supplementary Figures**

**
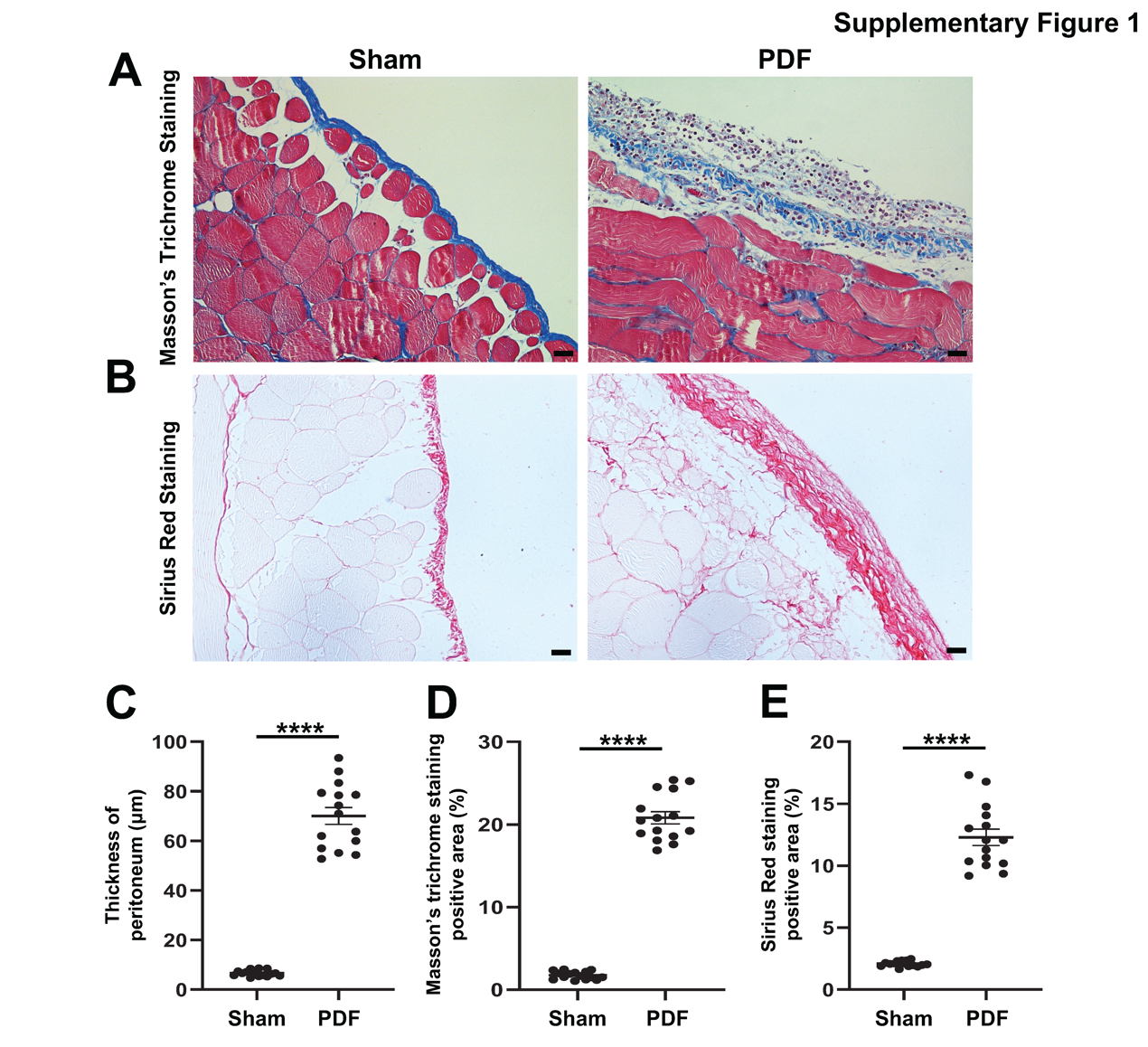
**

**Supplementary Figure 1** **Histopathological examination in a murine model of peritoneal fibrosis induced by 100 ml/kg 4.25% HG-PDF**

**(A)** Photomicrographs showed Masson’s trichrome staining of the peritoneum. **(B)** Photomicrographs showed Sirius red staining of the peritoneum. **(C)** Thickness of Masson-positive submesothelial area. **(D)** Area of the Masson-positive submesothelial area (%). **(E)** Area of Sirius red-positive submesothelial area (%). Data are expressed as mean ± SEM. *****P*<0.0001 versus Sham group. All scale bars = 20 μm.


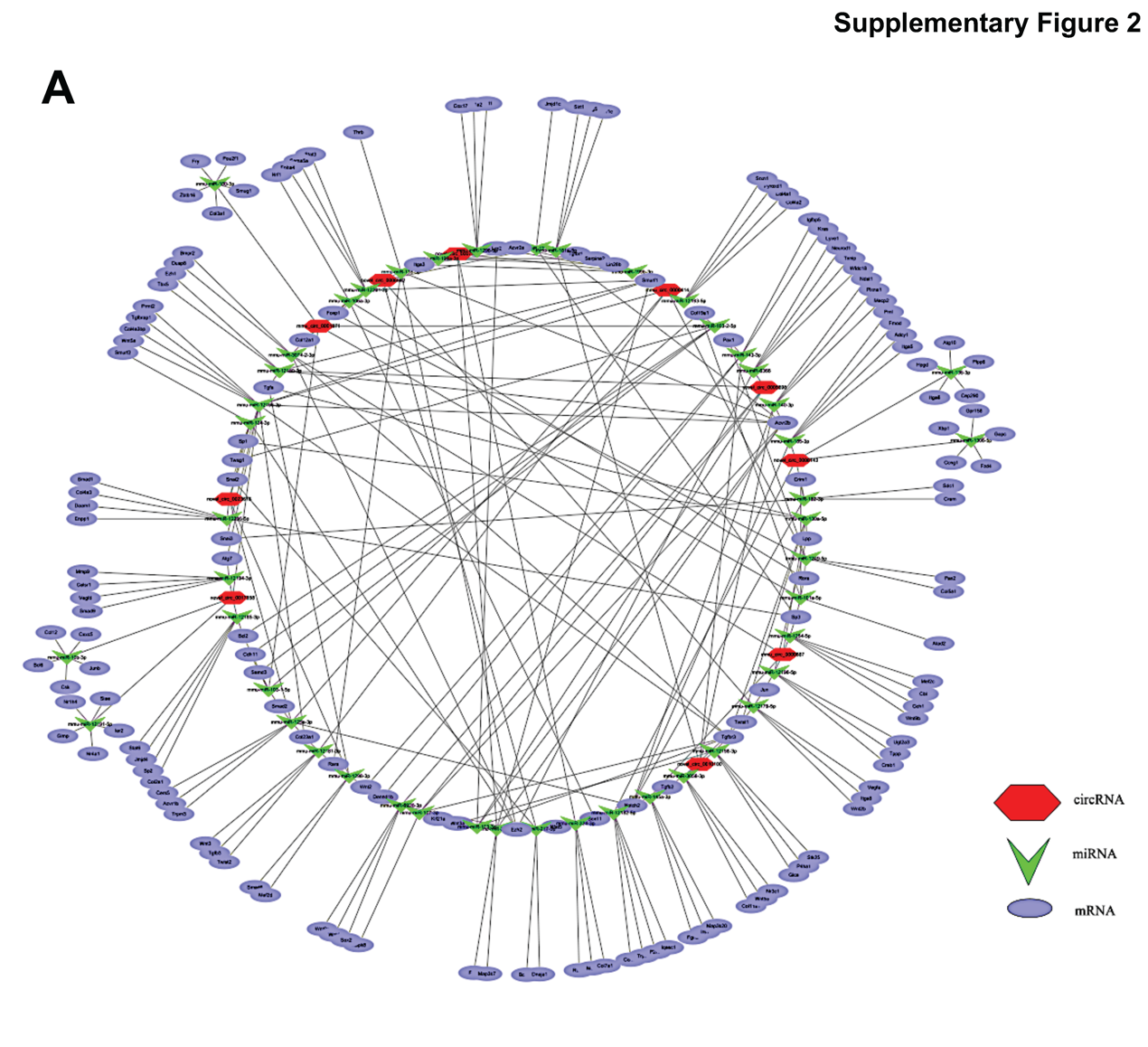


**Supplementary Figure 2 The interaction network of circRNA-miRNA-mRNA**

**(A)** The top 10 up-regulated circRNAs were selected to construct the circRNA-miRNA-mRNA network by Cytoscape software. For each circRNA, five miRNAs most likely binding to the circRNA and top 5 target genes to every miRNA were predicted.


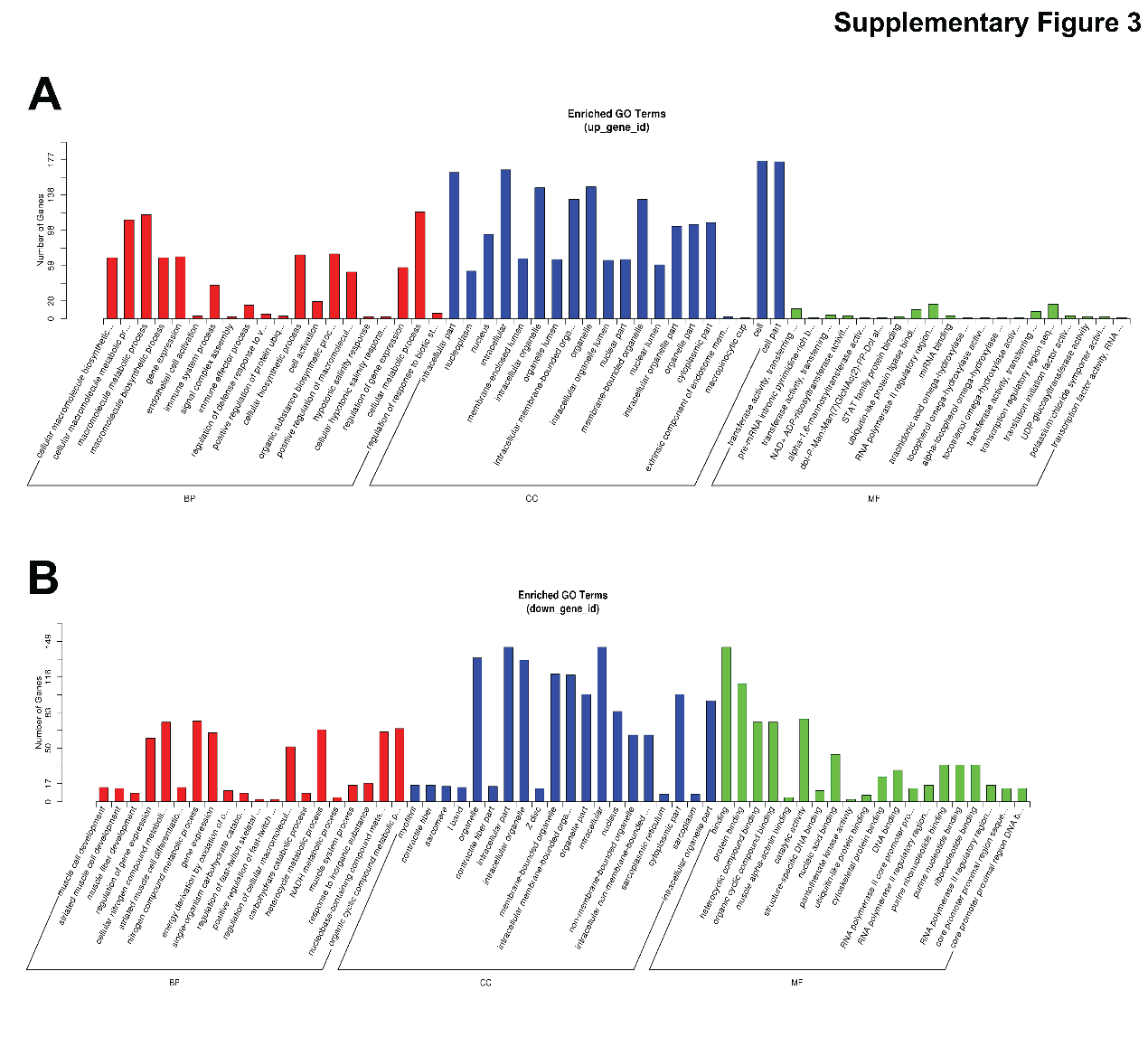


**Supplementary Figure 3 Gene ontology enrichment of circRNAs**

Gene ontology enrichment of up-regulated circRNAs **(A)** and down-regulated circRNAs **(B)** in biological functions, mainly includes biological process (BP), cell component (CC) and molecular function (MF).


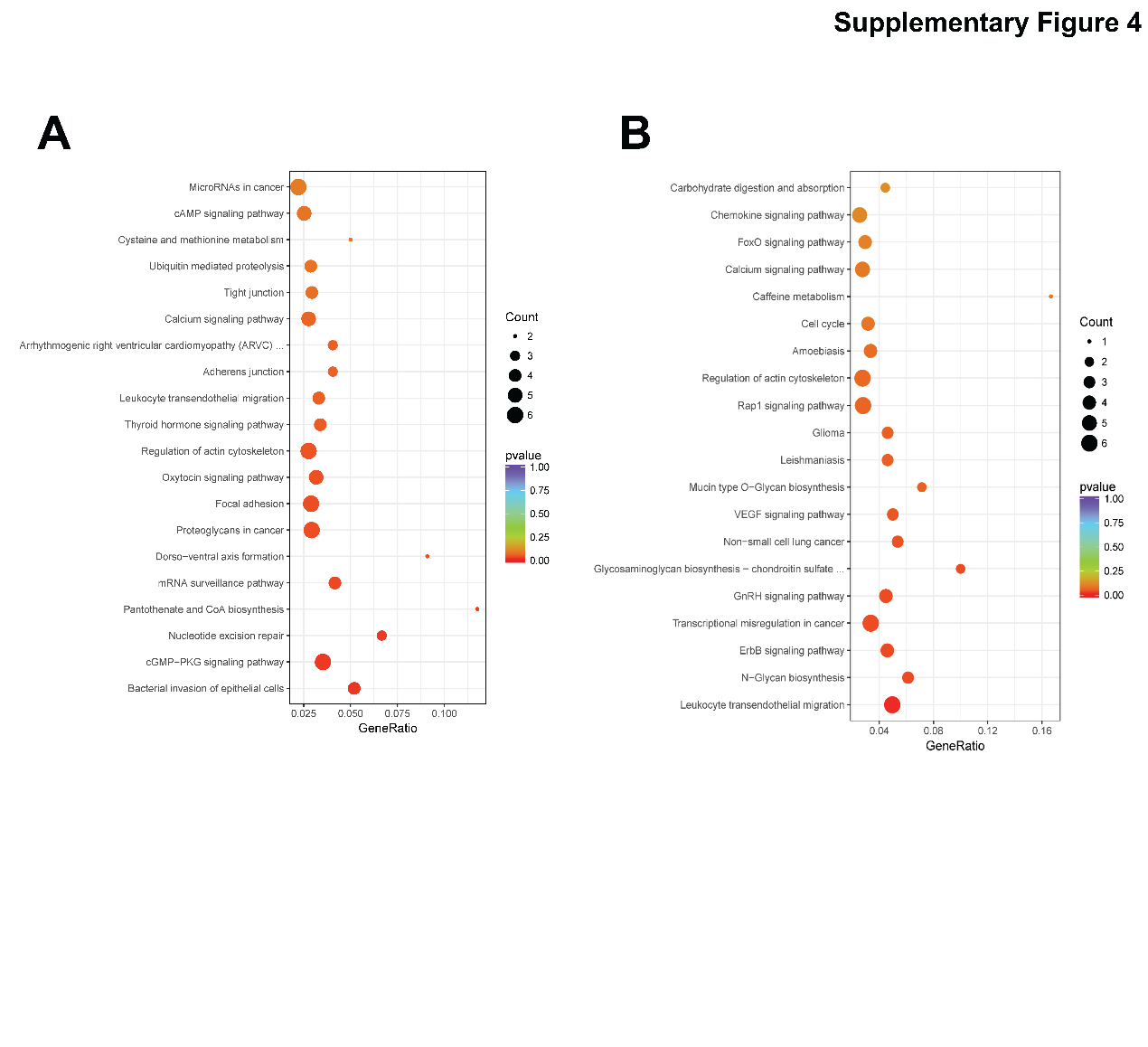


**Supplementary Figure 4 KEGG pathway enrichment of circRNAs**

The top of 20 KEGG enriched signaling pathways of down-regulated circRNAs **(A)** and up-regulated circRNAs **(B)**.


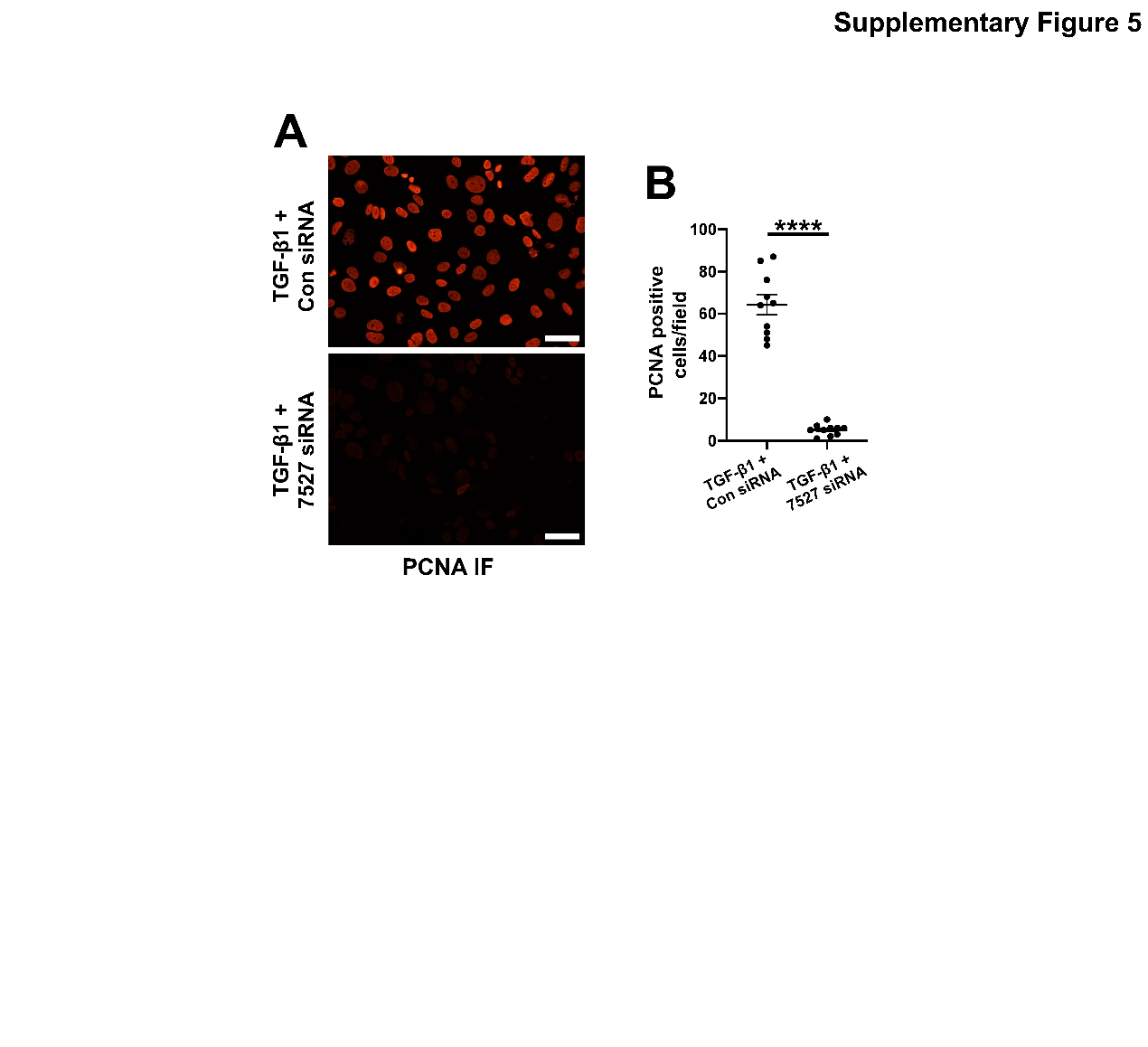


**Supplementary Figure 5 Photomicrographs illustrating immunofluorescence staining of PCNA**

**(A)** Photomicrographs illustrating immunofluorescence staining of PCNA. **(B)** Positive cells/fields of PCNA were quantitatively analyzed. Data are expressed as mean ± SEM. *****P*<0.0001 with the comparisons labeled. All scale bars = 50 μm.

**3. Supplementary Tables**

Supplementary Table 1. Biological information of the top 10 up-regulated circRNAs in peritoneal fibrosis

| CircRNAs ID | Chr number | Starting site | Termination site | Full-length | log2Fold Change | p value | padj | Source gene |
| --- | --- | --- | --- | --- | --- | --- | --- | --- |
| novel_circ_0007527 | chr16 | 20656542 | 20657609 | 1067 | 4.8457 | 7.31E-32 | 3.77E-28 | Psmd2 |
| novel_circ_0005698 | chr14 | 30346797 | 30350949 | 4152 | 2.7112 | 4.40E-10 | 1.14E-06 | Cacna1d |
| mmu_circ_0000687 | chr16 | 35929302 | 35931590 | 2288 | 1.3626 | 2.66E-07 | 0.000343 | Dtx3l |
| novel_circ_0008143 | chr16 | 92688975 | 92695737 | 6762 | 2.4058 | 2.33E-07 | 0.000343 | Runx1 |
| novel_circ_0006982 | chr15 | 78296975 | 78298378 | 1403 | 1.7394 | 7.41E-07 | 0.000637 | Csf2rb2 |
| novel_circ_0010100 | chr19 | 11475273 | 11480399 | 5126 | 2.231 | 1.05E-06 | 0.000658 | Ms4a6c |
| novel_circ_0017858 | chr5 | 120871128 | 120879332 | 8204 | 2.3111 | 1.15E-06 | 0.000658 | - |
| novel_circ_0023616 | chr9 | 21535907 | 21540623 | 4716 | 2.2689 | 9.02E-07 | 0.000658 | AB124611 |
| mmu_circ_0001871 | chrX | 47811157 | 47887198 | 76041 | 1.8505 | 1.29E-05 | 0.006074 | Smarca1 |
| mmu_circ_0000414 | chr12 | 107989464 | 107989830 | 366 | 2.0057 | 2.39E-05 | 0.009574 | Bcl11b |

Supplementary Table 2. Biological information of the top 10 down-regulated circRNAs in peritoneal fibrosis

| CircRNAs ID | Chr number | Starting site | Termination site | Full-length | log2Fold Change | p value | padj | Source gene |
| --- | --- | --- | --- | --- | --- | --- | --- | --- |
| novel_circ_0007895 | chr16 | 5171860 | 5173166 | 1306 | -0.93592 | 3.61E-07 | 0.000373 | Sec14l5 |
| novel_circ_0023883 | chr9 | 48717282 | 48743561 | 26279 | -1.1211 | 1.79E-06 | 0.000925 | Zbtb16 |
| novel_circ_0003608 | chr12 | 3835026 | 3873428 | 38402 | -0.82581 | 5.54E-05 | 0.012439 | Dnmt3a |
| novel_circ_0002224 | chr11 | 59122619 | 59124796 | 2177 | -1.7093 | 0.000266 | 0.041567 | Obscn |
| novel_circ_0017331 | chr4 | 82498237 | 82498769 | 532 | -1.7091 | 0.000266 | 0.041567 | Nfib |
| novel_circ_0000463 | chr10 | 33139073 | 33158278 | 19205 | -1.165 | 0.000349 | 0.049889 | Trdn |
| novel_circ_0005819 | chr14 | 34566856 | 34571921 | 5065 | -1.4362 | 0.000358 | 0.049889 | Ldb3 |
| novel_circ_0007152 | chr15 | 89242159 | 89246340 | 4181 | -1.1179 | 0.000448 | 0.059168 | Ppp6r2 |
| mmu_circ_0000906 | chr18 | 82664837 | 82682287 | 17450 | -0.83247 | 0.000537 | 0.06452 | Zfp236 |
| novel_circ_0023001 | chr8 | 85875633 | 85879810 | 4177 | -0.53519 | 0.000600 | 0.06761 | Phkb |

Supplementary Table 3. The potential complementary binding miRNAs for top 10 up-regulated circRNAs

| circRNA | miRNA1 | miRNA2 | miRNA3 | miRNA4 | miRNA5 |
| --- | --- | --- | --- | --- | --- |
| novel_circ_0007527 | mmu-miR-1298-5p | mmu-miR-199a-3p | mmu-miR-199b-3p | mmu-miR-20a-3p | mmu-miR-324-3p |
| novel_circ_0005698 | mmu-miR-103-3p | mmu-miR-107-3p | mmu-miR-6928-3p | mmu-miR-6366 | mmu-miR-12180-3p |
| mmu_circ_0000687 | mmu-miR-12196-5p | mmu-miR-1264-5p | mmu-miR-130a-5p | mmu-miR-145a-3p | mmu-miR-3074-2-3p |
| novel_circ_0008143 | mmu-miR-12182-5p | mmu-miR-1306-5p | mmu-miR-136-3p | mmu-miR-182-3p | mmu-miR-185-3p |
| novel_circ_0006982 | mmu-miR-12179-5p | mmu-miR-138-5p | mmu-miR-12201-3p | mmu-miR-217-5p | mmu-miR-1271-5p |
| novel_circ_0010100 | mmu-miR-12198-3p | mmu-miR-140-3p | mmu-miR-143-3p | mmu-miR-181a-5p | mmu-miR-3058-3p |
| novel_circ_0017858 | mmu-miR-10b-3p | mmu-miR-12185-3p | mmu-miR-12191-5p | mmu-miR-12194-3p | mmu-miR-12196-3p |
| novel_circ_0023616 | mmu-miR-12196-3p | mmu-miR-12206-5p | mmu-miR-124-3p | mmu-miR-125a-3p | mmu-miR-1298-3p |
| mmu_circ_0001871 | mmu-miR-100-3p | mmu-miR-101a-5p | mmu-miR-103-1-5p | mmu-miR-103-2-5p | mmu-miR-106a-3p |
| mmu_circ_0000414 | mmu-miR-12181-3p | mmu-miR-12193-5p | mmu-miR-12196-3p | mmu-miR-1249-5p | mmu-miR-125a-3p |

Supplementary Table 4. Target gene prediction of each miRNA

| miRNA | predicted target genes |
| --- | --- |
| mmu-miR-1298-5p | Egf, Lrp2, Nkd1, Cyp1a2, Cox17 |
| mmu-miR-199a-3p | Acvr2a, Itga3, Serpine2, Lin28b, Lrp2 |
| mmu-miR-199b-3p | Itga3, Lin28b, Serpine2, Lrp2, Acvr2a |
| mmu-miR-20a-3p | Ezh2, Fgf7, Smurf1, Map3k7, Acvr2b |
| mmu-miR-324-3p | Nfat5, Rtkn2, Mthfr, Sox11, Col7a1 |
| mmu-miR-103-3p | Tgfbr3, Acvr2b, Wnt3a, Lrp2, Kif21a |
| mmu-miR-107-3p | Kif21a, Tgfbr3, Mapk8, Acvr2b, Wnt3a |
| mmu-miR-6928-3p | Ezh2, Wnt9a, Wnt1, Dennd1b, Sox2 |
| mmu-miR-6366 | Ezh2, Wfdc18, Txnip, Neurod1, Lyve1 |
| mmu-miR-12180-3p | Ezh1, Acvr2b, Tgfa, Tbx5, Smurf1 |
| mmu-miR-12196-5p | Jun, Creb1, Tppp, Ugt2a3, Lpp |
| mmu-miR-1264-5p | Wnt9b, Sp3, Gch1, Cbl, Mef2c |
| mmu-miR-130a-5p | Twist1, Sp1, Lpp, Tgfa, Tgfbr3 |
| mmu-miR-145a-3p | Fgfr2, Tgfb2, Notch2, Irs1, Map3k20 |
| mmu-miR-3074-2-3p | Ezh2, Col12a1, Tgfbr3, Bmpr2, Dusp8 |
| mmu-miR-12182-5p | Sox11, Ccnd1, Trpc6, P2rx4, Iqsec1 |
| mmu-miR-1306-5p | Xbp1, Ccng1, Gpr158, Fzd4, Gopc |
| mmu-miR-136-3p | Hpgd, Itga6, Atg10, Cep290, Plpp6 |
| mmu-miR-182-3p | Snai3, Notch2, Crem, Crim1, Sdc1 |
| mmu-miR-185-3p | Itga5, Notch2, Adcy1, Fmod, Pml |
| mmu-miR-12179-5p | Wnt2b, Jun, Twist1, Itga8, Vegfa |
| mmu-miR-138-5p | Ezh2, Rara, Itga3, Acvr2b, Thrb |
| mmu-miR-12201-3p | Smurf1, Stat3, Sema5a, Col12a1, Epha4 |
| mmu-miR-217-5p | Ezh2, Snai2, Nfat5, Bcl11a, Dnaja1 |
| mmu-miR-1271-5p | Tgfbr1, Itga3, Sp3, Acvr2a, Jmjd1c |
| mmu-miR-12198-3p | Tgfbr3, Wnt3a, Glce, P4ha1, Stk25 |
| mmu-miR-140-3p | Notch2, Acvr2b, Mecp2, Plxna1, Ndst1 |
| mmu-miR-143-3p | Pax1, Wnt2, Kras, Igfbp5, Dennd1b |
| mmu-miR-181a-5p | Tgfbr1, Acvr2b, Acvr1c, Atg5, Sirt1 |
| mmu-miR-3058-3p | Col11a1, Twist1, Tgfb2, Wnt5b, Nr3c1 |
| mmu-miR-10b-3p | Junb, Cxxc5, Csk, Ccl12, Bcl6 |
| mmu-miR-12185-3p | Bcl2, Stat6, Jmjd4, Sp2, Col2a1 |
| mmu-miR-12191-5p | Ier2, Siae, Nr4a1, Nr1h4, Glmp |
| mmu-miR-12194-3p | Atg7, Mmp9, Celsr1, Vegfd, Smad9 |
| mmu-miR-12196-3p | Prmt2, Col4a3bp, Snai3, Smurf1, Wnt5a |
| mmu-miR-12196-3p | Tgfbrap1, Atg7, Tgfbr3, Col23a1, Acvr2b |
| mmu-miR-12206-5p | Smad1, Sp3, Col4a3, Daam1, Enpp1 |
| mmu-miR-124-3p | Snai2, Ezh2, Sp1, Twsg1, Smurf2 |
| mmu-miR-125a-3p | Col23a1, Notch2, Smad2, Cers5, Trpm3 |
| mmu-miR-1298-3p | Wnt2, Rara, Smad6, Tgfa, Mef2d |
| mmu-miR-100-3p | Col3a1, Smug1, Zbtb16, Pou2f1, Fry |
| mmu-miR-101a-5p | Rora, Sp3, Col19a1, Crim1, Atad2 |
| mmu-miR-103-1-5p | Samd3, Bcl2, Smad2, Col19a1, Cdh11 |
| mmu-miR-103-2-5p | Cdh11, Samd3, Bcl2, Twsg1, Col19a1 |
| mmu-miR-106a-3p | Col12a1, Nrf1, Foxp1, Sox11, Rora |
| mmu-miR-12181-3p | Wnt3, Tgfb3, Col23a1, Rara, Twist2 |
| mmu-miR-12193-5p | Col4a2, Smurf1, Col4a1, Pyroxd1, Srxn1 |
| mmu-miR-12196-3p | Col4a3bp, Smurf1, Wnt5a, Atg7, Tgfbr3 |
| mmu-miR-1249-5p | Col5a1, Rora, Pax1, Pax2, Foxp1 |
| mmu-miR-125a-3p | Col23a1, Notch2, Acvr1b, Smad2, Trpm3 |
